# Effects of a novel *E. coli* phytase expressed in *Pseudomonas fluorescens* on growth, bone mineralization, and nutrient digestibility in pigs fed corn–soybean meal diets

**DOI:** 10.1093/tas/txaa201

**Published:** 2020-11-04

**Authors:** Ping Ren, Laia Blavi, Caroline González-Vega, Yanhong Liu, Deana Hancock, Mercedes Vazquez-Añón, Ferdinando N Almeida, Hans H Stein

**Affiliations:** 1 Novus International, Inc., St. Charles, MO, USA; 2 Department of Animal Sciences, University of Illinois, Urbana, IL

**Keywords:** amino acid, bone ash, growth performance, novel *E. coli* phytase, pigs, phosphorus

## Abstract

Two studies were conducted to determine the effects of a novel *Escherichia coli* phytase expressed in *Pseudomonas fluorescens* on growth performance, bone mineralization, and nutrient digestibility in pigs fed corn-soybean meal diets. In experiment 1, 160 nursery pigs (9.79 ± 1.22 kg) were randomly allotted to one of four treatments with 10 pens per treatment and four pigs per pen. Phase I and phase II diets were provided from d 0 to d 14 and d 14 to d 28, respectively. Treatments included: positive control (PC) with all nutrients meeting requirements; negative control (NC) with standardized total tract digestible (STTD) P reduced by 0.15% and 0.14% compared with PC in phase I and phase II, respectively; and NC diets containing 250 or 500 units of phytase (FTU) per kilogram. Results demonstrated that pigs fed PC had greater (*P* < 0.01) ADG and G:F for the overall experimental period, and greater (*P* < 0.01) bone ash and P concentrations, compared with pigs fed NC or diets with phytase supplementation. Pigs fed diets containing phytase had greater (*P* < 0.01) ADG and G:F for the overall experimental period compared with pigs fed the NC diet without phytase, and bone ash and P weights were increased (*P* < 0.01) as well. In experiment 2, 63 growing barrows (56.25 ± 2.54 kg) were blocked by BW and randomly allotted to one of seven treatments with nine pens per treatment and one pig per pen. A basal corn–soybean meal diet was formulated to meet nutrient requirements for growing pigs with the exception that STTD P was reduced by 0.18% compared with the requirement, and Ca was included to achieve a Ca:STTD P ratio of 2.15. Six additional diets were formulated by adding 250, 500, 750, 1,000, 1,500, or 2,000 FTU/kg of phytase to the basal diet. Pigs were fed experimental diets for 12 d with 7 d of adaptation and 5 d of fecal sample collection. Results indicated that there was a linear (*P* < 0.01) increase in apparent total tract digestibility of ash and ether extract, and STTD of Ca and P also increased (linear, *P* < 0.05) in response to increasing doses of phytase. Increasing phytase levels in the diets resulted in increase (quadratic, *P* < 0.05) in apparent ileal digestibility of Arg, His, Ile, Lys, Trp, Asp, and Glu. In conclusion, the novel *E. coli* phytase was effective in increasing growth performance, bone mineralization, and Ca and P digestibility in pigs fed corn–soybean meal-based diets. Results also indicated that this phytase had the potential to enhance the digestibility of fat and certain AA.

## INTRODUCTION

Phosphorus in plant ingredients is poorly digested and utilized by pigs and poultry because approximately two-thirds of P in feedstuffs of plant origin is present in the phytate form (Ravindran et al., 1994), which is largely undigested in the gastrointestinal tract due to a lack of significant quantities of endogenous phytase (Viveros et al., 2000). Therefore, inorganic P has traditionally been used in diet formulations to meet the P requirement of pigs and poultry. However, since the first commercial phytase was launched in 1991 (Lei et al., 2013), exogenous phytase has been widely used in swine and poultry diets to reduce cost and P excretion from the animals.

Microbial phytases can be divided into three categories based on the position on the inositol ring where they initiate hydrolysis, which are the 3-, 5-, or 6-positions. Currently, both 3-phytases (*Aspergillus niger*) and 6-phytases (*E. coli, Peniophora lycii, Citrobacter braakii,* and *Buttiauxella* spp.) are approved for use in diets fed to pigs and poultry (Selle and Ravindran, 2007; Dersjant-Li et al., 2015). However, efficacy among microbial phytases may vary, and the efficacy depends on phytate-substrate affinity, resistance to proteases, and optimal pH (Dersjant-Li et al., 2015). Therefore, development of novel microbial phytases aims at achieving improved resistance to endogenous enzymes and greater activity over a wider range of pH.

Recently, a novel *E. coli.* phytase (CIBENZA PHYTAVERSE G10, Novus International, Inc., St. Charles, MO) expressed in *Pseudomonas fluorescens* was developed through Gene Site Saturated Mutagenesis (Garrett et al., 2004) and Gene Reassembly Evolution Technology (Solbak et al., 2005). This technology is expected to result in synergistic combinations of point mutations and therefore more efficient release of phytate-bound P. Indeed, in vitro models demonstrated that this novel *E. coli* phytase was more effective in hydrolyzing phytate-bound P at low substrate concentrations and with the presence of simulated gastric fluid, compared with the wild type *E. coli* phytase (Pieniazek et al., 2017). In vivo studies also demonstrated that this novel *E. coli* phytase resulted in improved growth performance, bone mineralization, and ileal amino acid digestibility in broilers (Pieniazek et al., 2017), as well as increased P digestibility in growing pigs (Almeida et al., 2017). However, no data for the effects of this phytase on growth performance, bone mineralization, and nutrient digestibility in pigs fed corn–soybean meal-based diets have been reported. Therefore, two experiments were conducted to test the hypotheses that 1) growth performance, bone mineralization, and nutrient digestibility will increase if this phytase is added to a corn–soybean meal-based diet and fed to pigs; and 2) that there is an optimal dose of phytase that maximizes Ca and P digestibility in pigs fed corn-soybean meal-based diets.

## MATERIALS AND METHODS

The Institutional Animal Care and Use Committee at the University of Illinois reviewed and approved the protocol for the first experiment. Novus International, Inc. Animal Ethical Committee reviewed and approved the protocol for the second experiment.

### Experiment 1

This experiment was conducted at Swine Research Center at the University of Illinois, Urbana-Champaign, IL. One hundred and sixty Line 359 × F46 nursery pigs [initial body weight (BW): 9.79 ± 1.22 kg; PIC, Hendersonville, TN, USA] were allotted to one of four treatments with 10 replicate pens per treatment and four pigs per pen in a randomized complete block design with initial BW as the blocking factor. Pen was the experimental unit and there were two barrows and two gilts in each pen. The Experimental Animal Allotment Program (Kim and Lindemann, 2007) was used to allot pigs to experimental diets. Pigs were housed in pens with fully slatted plastic floors and room temperature was controlled (27 ºC in week 1, 26 ºC in week 2, and 24 ºC in weeks 3 and 4).

The experiment was conducted over a 28 d-period and divided into two phases with phase I being from d 0 to d 14 and Phase II being from d 14 to d 28. In each phase, four diets were used (Tables 1 and 2). The positive control diets for both phases were corn-soybean meal-based diets formulated to meet or exceed requirements of all nutrients (NRC, 2012). A negative control diet was also formulated for each phase and this diet was similar to the positive control diet except that standardized total tract digestible (STTD) P was reduced by 0.15% and 0.14% in phase I and phase II, respectively. Two additional diets in each phase were formulated by adding 250 or 500 FTU/kg of the novel *E. coli* phytase to the negative control diet. All diets were formulated to contain 0.80 and 0.70% Ca in phase I and phase II, respectively. All experimental diets were fed in mash form.

**Table 1.** Ingredient composition (as-is basis) of experimental diets (experiment 1), phases I and II^*a*^

	Phase I				Phase II			
Ingredients, %	PC^*b*^	NC^*b*^	NC + 250 FTU^*c*^	NC + 500 FTU	PC	NC	NC + 250 FTU	NC + 500 FTU
Ground corn	60.24	60.66	60.66	60.66	61.79	62.19	62.19	62.19
Soybean meal, 48% CP^*d*^	33.80	33.80	33.80	33.80	33.00	33.00	33.00	33.00
Soybean oil	2.10	2.10	2.10	2.10	1.80	1.80	1.80	1.80
Limestone	1.14	1.52	1.52	1.52	1.10	1.44	1.44	1.44
Monocalcium phosphate	1.03	0.23	0.23	0.23	0.81	0.07	0.07	0.07
L-Lys HCl, 78% Lys	0.340	0.340	0.340	0.340	0.245	0.245	0.245	0.245
MHA^*e*^	0.225	0.225	0.225	0.225	0.170	0.170	0.170	0.170
L-Thr	0.125	0.125	0.125	0.125	0.085	0.085	0.085	0.085
Sodium chloride	0.60	0.60	0.60	0.60	0.60	0.60	0.60	0.60
Vitamin mineral premix^*f*^	0.30	0.30	0.30	0.30	0.30	0.30	0.30	0.30
Phytase premix^*g*^	0.10	0.10	0.10	0.10	0.10	0.10	0.10	0.10

^*a*^Phase I diets were fed to pigs from d 0 to 14 of the experiment, and phase II diets were fed from d 14 to d 28 of the experiment.

^*b*^PC, positive control; NC, negative control.

^*c*^FTU, phytase units per kg of complete diet.

^*d*^CP, crude protein.

^*e*^MHA is dry calcium salt of d,l-2-hydroxy-4-(methylthio)butanoic acid (84% Met activity, MHA, Novus International, Inc., St. Charles, MO).

^*f*^The vitamin-micromineral premix provided the following quantities of vitamins and micro minerals per kilogram of complete diet: Vitamin A as retinyl acetate, 11,136 IU; vitamin D_3_ as cholecalciferol, 2,208 IU; vitamin E as dl-alpha tocopheryl acetate, 66 IU; vitamin K as menadione dimethylprimidinol bisulphite, 1.42 mg; thiamine as thiamine mononitrate, 0.24 mg; riboflavin, 6.59 mg; pyridoxine as pyridoxine hydrochloride, 0.24 mg; vitamin B_12_, 0.03 mg; d-pantothenic acid as d-calcium pantothenate, 23.5 mg; niacin, 44.1 mg; folic acid, 1.59 mg; biotin, 0.44 mg; Cu, 20 mg as copper sulphate and copper chloride; Fe, 126 mg as ferrous sulphate; I, 1.26 mg as ethylenediamine dihydriodide; Mn, 60.2 mg as manganese sulphate; Se, 0.3 mg as sodium selenite and selenium yeast; and Zn, 125.1 mg as zinc sulphate.

^*g*^The phytase premix was prepared by mixing ground wheat with phytase (CIBENZA PHYTAVERSE G10, Novus International, Inc., St. Charles, MO) to provide 250 or 500 FTU/kg in complete diets in phase I or phase II, respectively.

**Table 2.** Analyzed nutrient compositions of experimental diets (experiment 1), phase I and phase II^*a*^

	Phase I				Phase II			
Analyzed values	PC^*b*^	NC^*b*^	NC + 250 FTU^*c*^	NC + 500 FTU	PC	NC	NC + 250 FTU	NC + 500 FTU
Dry matter, %	85.61	85.36	85.81	85.37	85.36	85.17	84.62	84.58
Ash, %	5.85	5.68	5.40	6.02	6.25	5.64	5.85	3.96
Crude protein, %	18.31	19.24	19.81	19.57	21.50	20.45	19.76	19.97
Ca, %	0.82	0.83	0.77	0.75	0.65	0.75	0.71	0.67
P, %	0.56	0.40	0.41	0.41	0.52	0.38	0.36	0.34
Amino acids, %								
Ile	0.78	0.81	0.85	0.92	0.78	0.79	0.80	0.86
Leu	1.60	1.67	1.74	1.85	1.59	1.66	1.67	1.74
Lys	1.34	1.35	1.37	1.46	1.31	1.28	1.27	1.39
Met	0.28	0.28	0.30	0.31	0.28	0.28	0.28	0.30
Thr	0.77	1.00	0.84	0.87	0.72	0.75	0.80	0.81
Val	0.94	0.95	1.02	1.08	0.94	0.96	0.96	1.02
Ala	0.94	0.96	1.02	1.06	0.92	0.96	0.98	1.02
Asp	1.99	2.04	2.12	2.27	1.94	1.94	2.03	2.15
Cys	0.32	0.29	0.31	0.32	0.28	0.29	0.30	0.30
Glu	3.31	3.50	3.59	3.86	3.24	3.37	3.48	3.62
Gly	0.79	0.85	0.90	0.94	0.76	0.83	0.87	0.91
Pro	1.16	1.20	1.27	1.32	1.17	1.18	1.21	1.23
MHA^*d*^	0.23	0.27	0.20	0.25	0.15	0.17	0.17	0.11

^*a*^Phase I diets were fed to pigs from d 0 to d 14 of the experiment and, Phase II diets were fed from d 14 to d 28 of the experiment.

^*b*^PC, positive control; NC, negative control.

^*c*^FTU, phytase units per kg of complete diet.

^*d*^MHA is dry calcium salt of d,l-2-hydroxy-4-(methylthio)butanoic acid (84% Met activity, MHA, Novus International, Inc., St. Charles, MO).

Pigs were allowed ad libitum access to feed and water throughout the experiment and all pigs were weighed on d 0, d 14, and d 28 of the experiment. The amount of feed offered was recorded daily and the amount of feed left in the feeder at the end of phase I and at the conclusion of the experiment was subtracted from the total amount of feed offered in each Phase. On the last day of the experiment, two pigs from each pen (one barrow and one gilt) were euthanized via captive bolt stunning. The right front foot was removed from these pigs and stored at –20 °C. Feet were autoclaved at 125 °C for 55 min and the third and fourth metacarpals were removed. The marrow of the broken metacarpals was removed and bones were dried and soaked in petroleum ether under a chemical hood for 72 h to remove the remaining marrow and fat. Bones were dried overnight at 130 °C and ashed at 600 °C for 16 h to calculate the concentration of bone ash.

### Experiment 2

The second experiment was conducted at Green Acres Animal Research and Testing Facility (Montgomery City, MO), which belongs to Novus International, Inc. A total of 63 TR4 × C22 growing barrows (BW = 56.25 ± 2.54 kg; PIC, Hendersonville, TN, USA) were used in this study. Pigs were housed individually in plastic-coated floor pens. Each barrow was tagged for individual identification. Daily amount of feed was calculated as three times the estimated requirement for metabolizable energy (197 kcal/BW^0.60^; NRC, 2012). Daily feed allowance was divided into two equal meals provided at 0600 and 1300 hours. Pigs had free access to water during the experimental period. Pigs were fed for 12 d, with the initial 7 d being the adaptation period, whereas fecal samples were collected during the following 5 d.

At the initiation of this study, pigs were weighed individually and allotted to one of the seven diets according to a randomized complete block design with initial BW being the blocking factor (Kim and Lindemann, 2007). There were nine pens per treatment and one pig per pen. The basal corn–soybean meal diet was formulated to meet nutrient requirements for growing pigs with the exception that STTD P was reduced by 0.18%, and Ca was included to achieve a Ca:STTD P ratio of 2.15 according to NRC (2012). Six additional diets were formulated by adding 250, 500, 750, 1,000, 1,500, or 2,000 FTU/kg this novel *E. coli* phytase to the basal diet (Tables 3 and 4).

**Table 3.** Ingredient composition and calculated nutrient profile of dietary treatments (experiment 2)

	Phytase inclusion levels, FTU^*a*^/kg						
Ingredients	0	250	500	750	1,000	1,500	2,000
Corn, yellow dent	71.86	71.66	71.66	71.66	71.66	71.66	71.66
Soybean meal, 47.5% CP	25.00	25.00	25.00	25.00	25.00	25.00	25.00
Choice white grease	1.00	1.00	1.00	1.00	1.00	1.00	1.00
Limestone	0.63	0.63	0.63	0.63	0.63	0.63	0.63
Salt	0.30	0.30	0.30	0.30	0.30	0.30	0.30
Vitamin premix^*b*^	0.05	0.05	0.05	0.05	0.05	0.05	0.05
Trace mineral premix^*b*^	0.25	0.25	0.25	0.25	0.25	0.25	0.25
Phytase premix^*c*^	0.00	0.20	0.20	0.20	0.20	0.20	0.20
TiO_2_	0.40	0.40	0.40	0.40	0.40	0.40	0.40
l-Lysine HCl	0.30	0.30	0.30	0.30	0.30	0.30	0.30
MHA^*d*^	0.08	0.08	0.08	0.08	0.08	0.08	0.08
l-Threonine	0.13	0.13	0.13	0.13	0.13	0.13	0.13
Total	100	100	100	100	100	100	100
Calculated nutrient composition							
ME^*e*^, kcal/kg	3390	3390	3390	3390	3390	3390	3390
CP^*e*^, %	18.33	18.33	18.33	18.33	18.33	18.33	18.33
SID^*f*^, %							
Lys	1.03	1.03	1.03	1.03	1.03	1.03	1.03
Thr	0.68	0.68	0.68	0.68	0.68	0.68	0.68
Met	0.33	0.33	0.33	0.33	0.33	0.33	0.33
Met + Cys	0.58	0.58	0.58	0.58	0.58	0.58	0.58
Trp	0.18	0.18	0.18	0.18	0.18	0.18	0.18
Ca, %	0.32	0.32	0.32	0.32	0.32	0.32	0.32
Total P, %	0.37	0.37	0.37	0.37	0.37	0.37	0.37
STTD^*g*^ P, %	0.15	0.15	0.15	0.15	0.15	0.15	0.15
Phytate P, %	0.24	0.24	0.24	0.24	0.24	0.24	0.24

^*a*^FTU, phytase units per kg complete diet.

^*b*^The vitamin premix supplied the following nutrients per kilogram of diet: vitamin A, 6,600 IU; vitamin D, 1,210 IU; vitamin E, 33 IU; vitamin K, 3.3 mg; riboflavin, 5.72 mg; niacin, 39.6 mg; pantothenic acid, 19.8 mg; vitamin B12, 26.4 µg; the trace mineral premix supplied the following nutrients per kilogram of diet: Zn, 120 mg as zinc sulphate; Cu, 12 mg as copper; Mn, 30 mg as manganese sulphate; Fe, 80 mg as ferrous sulphate; I, 0.4 mg as ethylenediamine dihydriodide; Se, 0.3 mg as sodium selenite.

^*c*^Six phytase premixes were prepared by mixing ground corn and phytase (CIBENZA PHYTAVERSE G10, Novus International, Inc., St. Charles, MO) to provide 250, 500, 750, 1,000, 1,500, or 2,000 FTU/kg in the complete diets, respectively.

^*d*^MHA is dry calcium salt of d,l-2-hydroxy-4-(methylthio)butanoic acid (84% Met activity, MHA, Novus International, Inc., St. Charles, MO).

^*e*^ME and CP represented metabolizable energy and crude protein, respectively.

^*f*^SID, standardized ileal digestible.

^*g*^STTD, standardized total tract digestible.

**Table 4.** Analyzed nutrient concentrations of dietary treatments (experiment 2)

	Phytase inclusion levels, FTU^*a*^/kg						
Items, %	0	250	500	750	1,000	1,500	2,000
Dry matter	87.05	86.94	87.04	87.16	87.06	87.07	87.07
Crude protein	19.36	18.85	19.27	17.53	18.54	19.76	19.93
Ether extract	3.26	3.13	3.03	3.50	3.31	2.72	3.07
Crude fiber	2.50	2.30	2.10	2.40	2.20	2.20	2.20
Ash	4.10	3.75	4.25	4.08	4.19	3.98	4.06
Gross energy, kcal/kg	3,916	4,288	3,912	3,926	3,919	3,925	3,916
Ca	0.36	0.34	0.34	0.36	0.36	0.33	0.37
P	0.37	0.38	0.35	0.37	0.35	0.35	0.36
Phytate	0.86	0.84	0.87	0.85	0.84	0.85	0.85
Phytate P^*b*^	0.24	0.24	0.25	0.24	0.24	0.24	0.24
Phytase, FTU/kg	55	310	618	993	1,322	1,653	2,263
Indispensable amino acids							
Arg	1.16	1.23	1.29	1.13	1.14	1.22	1.26
His	0.46	0.60	0.50	0.45	0.45	0.48	0.49
Ile	0.69	0.69	0.76	0.67	0.67	0.72	0.75
Leu	1.56	1.56	1.66	1.53	1.54	1.59	1.66
Lys	1.15	1.22	1.22	1.13	1.16	1.21	1.26
Met	0.30	0.31	0.32	0.29	0.29	0.31	0.31
Phe	0.88	0.93	0.97	0.87	0.87	0.92	0.96
Thr	0.80	0.84	0.88	0.78	0.80	0.82	0.92
Trp	0.15	0.16	0.17	0.16	0.15	0.16	0.14
Val	0.69	0.72	0.74	0.68	0.68	0.71	0.73
Dispensable amino acids							
Ala	0.97	1.00	1.02	0.94	0.95	0.98	1.01
Asp	1.77	1.88	1.99	1.73	1.74	1.86	1.95
Cys	0.30	0.32	0.33	0.30	0.30	0.31	0.32
Glu	3.19	3.36	3.48	3.11	3.14	3.31	3.45
Gly	0.74	0.77	0.81	0.72	0.73	0.77	0.79
Pro	0.84	0.85	0.87	0.81	0.81	0.85	0.87
Ser	0.90	0.95	0.99	0.88	0.89	0.94	0.98
Tyr	0.46	0.49	0.51	0.45	0.45	0.48	0.50

^*a*^ FTU, phytase units.

^*b*^Calculated as 28.2% of phytate (Tran and Sauvant, 2004).

Fecal samples were collected via grab sampling from each pig from d 7 to d 11. Fecal samples collected from the same pen during the 5 d were pooled and mixed thoroughly, and a subsample of 250 to 350 g feces from each pig was placed in a heated oven (NHP-PD-ECO, Win-Holt, Woodbury, NY, USA) at 65 °C for 15–18 h. The dried feces were ground using a rotor mill (Pulverisette 14, Fritsch GmbH, Idar-Oberstein, Germany) fitted with a 1 mm screen.

At the end of the experiment, all pigs were euthanized using captive bolt. Ileal digesta samples were collected from all pigs by gently squeezing the content from the last 1 m proximal to the ileal–cecum junction. Ileal digesta samples were lyophilized in a freeze dryer (Alpha 1–4 LSC, IMA Life, Bologna, Italy) for 5 d. The dried ileal digesta samples were ground to a particle size of 1 mm using a blender mill (Oster Pro 1200 Blender, Hilliard, OH).

### Chemical Analysis

Ground diets, feces, and ileal digesta were thoroughly homogenized and a subsample was collected for analyses. In experiment 1, corn and soybean meal were analyzed for phytic acid (Ellis and Morris, 1983). Diet samples, corn, and soybean meal were analyzed for dry matter by oven drying at 135 ºC for 2 h (Method 930.15; AOAC, 2007) and for ash (Method 942.05; AOAC, 2007). Diets were analyzed for N using the combustion procedure (Method 990.03; AOAC, 2007) on an Elementar Rapid N-cube protein/nitrogen apparatus (Elementar Americas Inc., Mt. Laurel, NJ). Crude protein was calculated as N × 6.25. Gross energy was analyzed in corn and soybean meal using an isoperibol bomb calorimeter (Model 6300, Parr Instruments, Moline, IL). Benzoic acid was used as the standard for calibration. Calcium and P were analyzed in bone ash, diets, corn, and soybean meal using inductively coupled plasma-optical emission spectrometry (ICP-OES; Method 985.01 A, B, and C; AOAC, 2007) after wet ash sample preparation (Method 975.03 B(b); AOAC, 2007). Diets were also analyzed for phytase activity (ISO, 2009) and for amino acids by cation exchange chromatography (method 994.12; AOAC, 2007). The concentration of methionine hydroxy analog (MHA, 2-hydroxy-4-methylthio-butanoic acid calcium salt; Novus International, Inc., St. Charles, MO) was analyzed in the diets following the procedures of Ontiveros et al. (1987).

In experiment 2, diets and fecal samples were analyzed for dry matter (method 934.01; AOAC, 2006), ether extract (method 920.39; AOAC, 2006), crude protein (method 984.13; AOAC, 2006), crude fiber (method 978.10; AOAC, 2006), ash (method 923.03; AOAC, 2006), Ca (method 984.27; AOAC, 2006), and P (method 984.27; AOAC, 2006). Phytase activity in the diets was analyzed according to ISO (2009). Amino acid (AA) concentrations of diets and ileal digesta were determined using Method 982.30 E (a,b,c) (AOAC, 2006). Samples were hydrolyzed in 6 *N* HCl for 24 h at 110 °C under nitrogen atmosphere. For Met and Cys, performic acid oxidation was carried out before acid hydrolysis. The AA in the hydrolysate were determined by HPLC after postcolumn derivatization. Titanium concentration in the diets and fecal samples was analyzed according to the procedures described by Myers et al. (2004).

### Calculations

The percentage unit of phytate-bound P in corn and soybean meal was calculated as 28.2% of phytate (Tran and Sauvant, 2004) and the concentration of nonphytate P was calculated by subtracting phytate-bound P from total P (Table 5).

**Table 5.** Analyzed nutrient composition of corn and soybean meal (as-fed basis)

	Corn	Soybean meal
GE^*a*^, kcal/kg	3,799	4,162
DM^*b*^, %	83.76	87.75
Ash, %	1.05	7.51
Phytate, %	0.77	1.60
P, %	0.27	0.65
Phytate bound P^*c*^, %	0.22	0.45
Non-phytate P^*d*^, %	0.05	0.20

^*a*^GE, gross energy.

^*b*^DM, dry matter.

^*c*^Calculated as 28.2% of phytate (Tran and Sauvant, 2004).

^*d*^Calculated as total P subtracting phytate-bound P.

Average daily gain (ADG), average daily feed intake (ADFI), and gain:feed (G:F) were calculated for each pen and treatment group in experiment 1. Bone ash percentage was calculated by dividing the quantity of bone ash by the quantity of fat-free dried bone multiplied by 100. The quantity of bone P in grams was calculated by multiplying the bone P percentage by the quantity of bone ash and dividing by 100. The average bone ash percentage of the 2 pigs in each pen was used for statistical analyses.

The apparent total tract digestibility (ATTD) of Ca and P in experiment 2 was calculated according to NRC (2012). The STTD of Ca and P were calculated by accounting for endogenous losses of Ca (330 mg/kg DM intake; Merriman and Stein, 2016) and P (190 mg/kg DM intake; NRC, 2012), respectively. Apparent ileal digestibility (AID) of each AA in all diets was calculated using the equation described by Stein et al. (2007).

### Statistical Analysis

Normality of residuals and identification of outliers were determined by the UNIVARIATE procedure of SAS (SAS Inst. Inc., Cary, NC). Pen was the experimental unit. The LSMEANS procedure was used to calculate the least square mean values. The pooled SEM was calculated for each measurement. A probability of *P* ≤ 0.05 was considered significant and 0.05 < *P* ≤ 0.1 was a trend.

In experiment 1, data for BW, ADG, ADFI, G:F, bone ash, and bone P were analyzed using the Proc GLM of SAS (SAS Inst. Inc., Cary, NC). Tukey–Kramer adjustment was used for multiple comparisons of the least square means. PDIFF option was used to separate the least square means.

In experiment 2, the GLIMMIX procedure was used to analyze data. Diet was considered the fixed effect, and block was the random effect. Polynomial orthogonal contrasts were used to determine linear, quadratic, and cubic effects of phytase on response variables. A broken-line linear ascending model (BLL), a broken-line quadratic ascending model (BLQ), and a quadratic polynomial model (QP) were fitted to the data for STTD P and STTD Ca to estimate the optimal dose of phytase to maximize these parameters (Gonçalves et al., 2016). Correlated data structure and heterogenous error variance were accounted for in all models. The best model was selected based on maximum likelihood-based Bayesian information criterion (BIC), with BIC values greater than two considered a significant improvement in fit (Milliken and Johnson, 2009). For the models with similar BIC values, the model with the narrowest 95% confidence interval (CI) was considered the best model.

## RESULTS

### Experiment 1

Corn contained 0.77% phytic acid, 0.22% phytate P, and 0.05% nonphytate P and soybean meal contained 1.60% phytic acid, 0.45% phytate P, and 0.20% nonphytate P (Table 5). Positive and negative control diets had negligible phytase activity, but the diets in which 250 or 500 FTU/kg of phytase were included contained the expected quantities of phytase (Table 6).

**Table 6.** Expected and analyzed activity of phytase in experimental diets (experiment 1)

	Activity		
Treatment	Expected	Analyzed	% Recovery
Phase I			
1. Positive control (PC)	0	<60	–
2. Negative control (NC)	0	<60	–
3. NC + 250 FTU/kg phytase	250	293	117
4. NC + 500 FTU/kg phytase	500	541	108
Phase II			
1. Positive control (PC)	0	<60	–
2. Negative control (NC)	0	<60	–
3. NC + 250 FTU/kg phytase	250	305	122
4. NC + 500 FTU/kg phytase	500	551	110

### Growth Performance

All pigs remained healthy and consumed their diets without apparent problems. Pigs fed NC and diets supplemented with 250 or 500 FTU/kg phytase had reduced (*P* < 0.01) ADG and G:F during phase I compared with pigs fed PC (Table 7). There were no differences between pigs fed NC and phytase diets in terms of ADG, ADFI, and G:F in phase I. There was no difference between pigs fed PC and the phytase diet with 500 FTU/kg in terms of ADFI in phase I. Pigs fed NC or diets containing 250 or 500 FTU/kg phytase had lower (*P* < 0.01) final BW, ADG, and G:F during phase II compared with pigs fed PC. Phytase supplementation at 250 or 500 FTU/kg increased (*P* < 0.01) ADG and G:F in Phase II compared with NC. Pigs fed the diet containing 500 FTU/kg phytase had greater (*P* < 0.05) BW on d 28 and greater (*P* < 0.05) ADFI in phase II than pigs fed NC. There were no differences between phytase treatments in terms of final BW on d 28 and ADG, ADFI, and G:F in phase II. Additionally, pigs fed NC and diets containing phytase at 250 or 500 FTU/kg had lower (*P* < 0.01) ADG and G:F during the entire experimental period compared with pigs fed PC. Pigs fed NC or diets with 250 FTU/kg phytase had lower (*P* < 0.01) ADFI during the entire experimental period than those fed PC, but pigs fed the diet containing phytase at 500 FTU/kg had ADFI during the entire experimental period that was not different from that of pigs fed PC. Pigs fed the diets containing phytase had greater (*P* < 0.01) ADG and G:F during the entire experimental period than pigs fed NC.

**Table 7.** Effect of phytase on growth performance of nursery pigs fed corn-SBM based diets^*a*^

	Dietary treatments					
Items	PC^*b*^	NC^*b*^	NC + 250 FTU^*c*^	NC + 500 FTU	SEM	*P*-value
Initial BW, kg	9.79	9.76	9.8	9.8	0.39	1.00
Phase I, d 0 to d 14						
ADG^*d*^, g/d	406^a^	300^b^	329^b^	343^b^	16.01	< 0.01
ADFI^*d*^, g/d	691^a^	584^b^	610^b^	634^ab^	28.04	0.02
G:F^*d*^, g/g	0.592^a^	0.514^b^	0.536^b^	0.541^b^	0.012	< 0.01
BW on d 14, kg	15.5	14.0	14.4	14.6	0.56	0.06
Phase II, d 14 to d 28						
ADG, g/d	574^a^	371^c^	423^b^	472^b^	17.43	< 0.01
ADFI, g/d	896^a^	746^c^	799^bc^	833^ab^	28.39	0.01
G:F, g/g	0.64^a^	0.498^d^	0.529^c^	0.566^b^	0.009	< 0.01
BW on d 28, kg	23.5^a^	19.2^c^	20.3^bc^	21.2^b^	0.68	< 0.01
Overall, d 0 to d 28						
ADG, g/d	490^a^	335^c^	376^b^	407^b^	12.71	< 0.01
ADFI, g/d	793^a^	665^b^	705^b^	733^ab^	23.94	< 0.01
G:F, g/g	0.617^a^	0.505^d^	0.533^c^	0.556^b^	0.008	< 0.01

^a–d^Within a row, means without a common superscript differ (*P* < 0.05).

^*a*^Data are LSMEANS of 10 observations per treatment.

^*b*^PC, positive control; NC, negative control.

^*c*^FTU, phytase units per kg complete diet.

^*d*^ADG, average daily gain; ADFI, average daily feed intake; G:F, gain:feed.

### Bone Mineralization

Pigs fed NC and diets containing 250 or 500 FTU/kg phytase had lower (*P* < 0.01) fat-free dried bone weight, bone ash and P amounts, and bone ash percentage compared with pigs fed PC (Table 8). Phytase supplementation at 250 or 500 FTU/kg increased (*P* < 0.01) fat-free dried bone weight, bone ash, and bone P amounts compared with NC. Phytase supplementation at 500 FTU/kg resulted in a greater (*P* < 0.01) bone ash percentage compared with NC.

**Table 8.** Effect of the novel *E. coli* phytase on bone mineralization of nursery pigs fed corn-SBM based diets^*a*^

	Dietary treatments					
Item	PC^*b*^	NC^*b*^	NC + 250 FTU^*c*^	NC + 500 FTU	SEM	*P*-value
Fat-free dried bone weight, g	4.02^a^	2.79^c^	3.16^b^	3.31^b^	0.13	< 0.01
Bone ash weight, g	2.13^a^	1.35^c^	1.57^b^	1.68^b^	0.07	< 0.01
Bone ash, %	52.98^a^	48.58^c^	49.71^bc^	50.7^b^	0.57	< 0.01
Bone P, g	0.72^a^	0.48^c^	0.55^b^	0.58^b^	0.02	< 0.01
Bone P, %	18.00^a^	17.23^b^	17.53^ab^	17.63^ab^	0.17	0.02

^a–d^Within a row, means without a common superscript differ (*P* < 0.05).

^*a*^Data are LSMEANS of 10 observations per treatment.

^*b*^PC, positive control; NC, negative control.

^*c*^FTU, phytase units per kg complete diet.

### Experiment 2

*Total tract digestibility of energy and nutrients.* Increasing phytase dosages resulted in linear (*P* < 0.01) increase in ATTD of ash, ether extract, Ca, and P and increased (linear, *P* < 0.01) STTD of Ca and P in response to increasing dosages of phytase (Table 9). The ATTD of dry matter (*P* < 0.05), crude fiber (*P* < 0.01), and energy (*P* < 0.01) responded in a quadratic manner to the increase in dietary phytase.

**Table 9.** Effect of increasing dosage of the novel *E. coli* phytase on total tract digestibility of energy and nutrients in growing pigs fed corn-soybean meal based diets

	Phytase inclusion levels, FTU^*a*^/kg								*P*-value			
Items	0	250	500	750	1,000	1,500	2,000	SEM	Diet	Linear	Quadratic	Cubic
ATTD^*b*^, %												
Ash	57.20	62.29	67.02	66.43	66.36	67.89	65.27	1.34	<0.01	<0.01	<0.01	0.13
DM	88.20	88.33	87.83	87.96	87.14	88.77	88.96	0.42	0.03	0.09	0.02	0.74
EE	45.63	49.62	48.00	51.45	45.64	57.54	58.30	3.16	<0.01	<0.01	0.38	0.98
CF	52.29	45.80	43.22	46.88	41.16	49.62	53.49	3.23	0.03	0.25	< 0.01	0.35
CP	87.24	87.50	86.96	87.30	86.10	88.24	87.60	0.64	0.3	0.43	0.35	0.52
GE	87.33	87.47	86.04	86.62	85.53	87.50	87.61	0.49	<0.01	0.43	<0.01	0.36
Ca	59.94	71.74	78.67	80.96	82.51	80.98	79.06	2.08	<0.01	<0.01	<0.01	0.01
P	40.25	56.04	61.95	69.25	68.46	73.20	72.36	2.65	< 0.01	<0.01	<0.01	0.05
STTD^c^, %												
Ca	67.92	79.73	86.65	88.94	90.49	88.96	87.05	2.08	<0.01	<0.01	<0.01	0.01
P	44.82	60.61	66.52	73.81	73.03	77.77	76.93	2.65	<0.01	<0.01	<0.01	0.05

^*a*^FTU, phytase units.

^*b*^ATTD, apparent total tract digestibility; DM, EE, CF, CP, and GE represented dry matter, ether extract, crude fiber, crude protein and gross energy, respectively.

^*c*^STTD, standardized total tract digestibility.

*Apparent ileal digestibility of amino acids*. Increasing phytase levels in the diets resulted in quadratic (*P* < 0.05) increases in AID of Arg, His, Ile, Lys, Trp, Asp, and Glu (Table 10). There were also tendencies (quadratic, *P* < 0.10) for AID of Phe, Thr, Val, Cys, Ser, and Tyr to increase with increased phytase levels in the diets.

**Table 10.** Effect of increasing dosage of the novel *E. coli* phytase on apparent ileal digestibility of amino acids in growing pigs fed corn-soybean meal-based diets

	Phytase inclusion levels, FTU^*a*^/kg								*P*-value			
Items	0	250	500	750	1,000	1,500	2,000	SEM	Diet	Linear	Quadratic	Cubic
Indispensable amino acids												
Arg	84.59	89.70	87.74	87.43	87.42	86.00	86.97	1.38	0.18	0.35	0.03	0.09
His	80.25	85.88	82.16	80.97	82.67	81.95	83.47	1.68	0.22	0.63	0.03	0.09
Ile	79.35	85.65	83.68	82.94	83.65	80.75	82.37	2.10	0.20	0.33	0.04	0.16
Leu	77.99	84.24	79.37	79.96	81.34	79.87	82.30	1.89	0.26	0.87	0.11	0.04
Lys	81.38	86.98	86.20	84.32	84.67	83.57	84.43	1.90	0.24	0.29	0.02	0.35
Met	77.28	83.31	80.30	79.95	81.15	77.10	78.95	2.68	0.39	0.73	0.13	0.20
Phe	77.44	83.85	80.35	80.19	81.36	77.25	82.26	2.06	0.17	0.74	0.09	0.11
Thr	69.88	78.03	74.97	74.24	74.93	71.99	73.58	2.97	0.37	0.46	0.07	0.17
Trp	63.82	74.74	71.18	71.33	70.58	69.34	72.14	2.48	0.07	0.11	0.02	0.04
Val	71.87	79.93	76.05	75.16	76.72	72.09	77.66	2.65	0.17	0.71	0.06	0.13
Dispensable amino acids												
Ala	77.93	81.13	76.73	77.51	80.61	74.15	79.84	2.46	0.37	0.48	0.61	0.26
Asp	72.93	81.07	79.47	78.42	78.56	77.13	77.57	1.98	0.06	0.08	0.01	0.11
Cys	63.02	74.47	69.98	68.60	69.97	65.41	64.96	4.18	0.31	0.55	0.07	0.19
Glu	75.80	84.12	82.54	81.55	81.49	76.62	77.97	2.41	0.08	0.17	0.04	0.21
Gly	58.46	69.03	62.78	64.48	64.71	62.93	59.35	4.47	0.69	0.68	0.31	0.18
Pro	74.55	79.47	70.55	73.24	69.67	71.32	62.80	4.52	0.25	0.41	0.80	0.24
Ser	72.02	80.35	76.99	77.09	77.74	74.31	75.83	2.79	0.27	0.35	0.08	0.11
Tyr	78.82	85.23	83.43	82.47	83.28	79.38	81.46	2.47	0.22	0.37	0.06	0.24

^*a*^FTU, phytase units.

**Figure 1.**
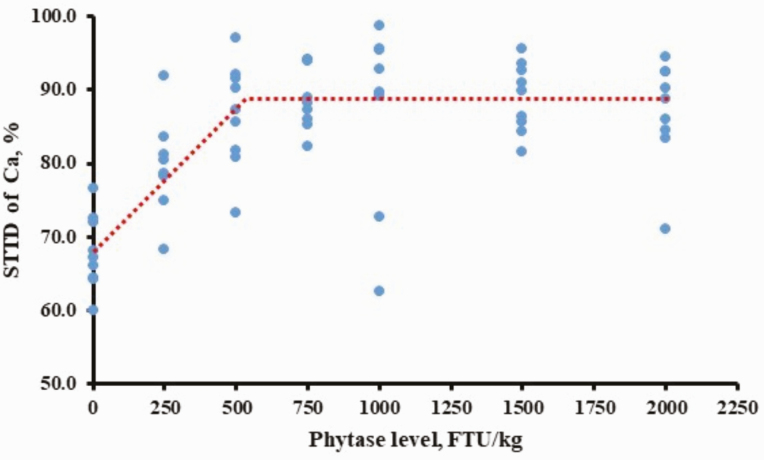
Fitted broken-line linear ascending model (BLL) for estimation of optimal dose of phytase on STTD of Ca in growing pigs fed corn-SBM diet. The BLL model estimated the maximum STTD of Ca at 526 phytase units (FTU)/kg diet (95% CI: [366, 686 FTU/kg]). The estimated equations were: STTD of Ca, % = 88.57 − 0.039 × (526 − phytase), if phytase < 526 FTU/kg; STTD of Ca = 88.57%, if phytase ≥ 526 FTU/kg.

*Estimation of optimal dosage of phytase on standardized total tract digestibility of Ca and P.* In terms of STTD of Ca, different combinations of heterogenous variances were compared with homogenous variance, which yielded two variance groups (“0, 750, 1,500” vs. “250, 500, 1,000, 2,000”; data not shown) having the lowest BIC value. The best-fitting model was BLL (BIC = 415.80; Figure 1), compared with BLQ (BIC = 417.80) and QP models (BIC = 430.17). The maximum STTD of Ca was obtained at 526 FTU/kg phytase (95% CI: [366, 686 FTU/kg]). The estimated equations for the BLL model were:

$$\begin{array}{ll} STTD\;of\;Ca=\;\; & 88.57-0.039\times \left(526\;\;-\;phytase\right),\;\\ & if\;phytase<526 \end{array}$$

$$\begin{array}{l} STTD\;of\;Ca=88.57,\;if\;phytase\geq 526 \end{array}$$

**Figure 2.**
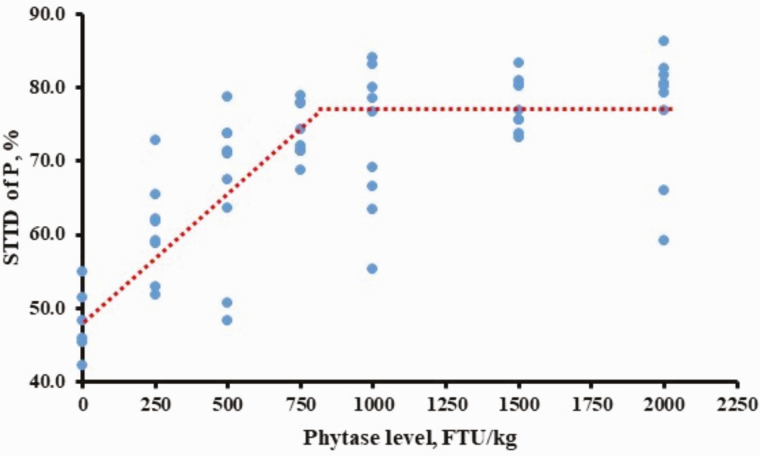
Fitted broken-line linear ascending model (BLL) for estimation of optimal dose of phytase on STTD of P in growing pigs fed corn–SBM diet. The BLL model estimated the maximum STTD of P at 834 phytase units (FTU)/kg phytase (95% CI: [727, 942 FTU/kg]). The estimated equations were: STTD of P, % = 77.10 – 0.035 × (834 − phytase), if phytase < 834 FTU/kg; STTD of P = 77.10%, if phytase ≥ 834 FTU/kg.

In terms of STTD of P, combinations of heterogenous variances were compared with homogenous variance, which resulted in two variance groups (“0, 250, 500, 1,000, 2,000” vs. “750, 1,500”; data not shown) having the lowest BIC value. The best-fitting model was BLL (BIC = 422.90; Figure 2), compared with BLQ (BIC = 422.40) and QP models (BIC = 432.38). The maximum STTD of P was obtained at 834 FTU/kg phytase (95% CI: [727, 942 FTU/kg]). The estimated equations for the BLL model were:

$$\begin{array}{ll} STTD\;of\;P=\;\; & 77.10-0.035\times \left(834\;\;-\;phytase\right),\;\;\\ & if\;phytase<834 \end{array}$$

$$\begin{array}{l} STTD\;of\;P=77.10,\;if\;phytase\geq 834 \end{array}$$

## DISCUSSION

### Effect of Phytase on Growth Performance and Bone Mineralization

The observation that phytase increased growth performance and bone mineralization was also observed in previous experiments (Kühn and Männner, 2012; Santos et al., 2014; Zeng et al., 2014). The improved growth performance and bone mineralization by phytase supplementation is mainly a result of increased P digestibility (Kies et al., 2006; Zeng et al., 2014; Adedokun et al., 2015), because if phytase makes more P available to pigs, growth performance and bone mineralization can be improved. Results of several studies indicated that supplementation of 500 FTU/kg phytase may restore growth performance and bone mineralization in pigs and chickens fed diets with reduced concentrations of P (Walk et al., 2013; Zeng et al., 2014; Truong et al., 2017). The reason 500 FTU/kg of phytase in the current study did not result in growth performance that was equivalent to pigs fed PC may be that the reductions in P (0.15 and 0.14% STTD P in phase I and II, respectively) in the NC and phytase treatments were greater than the total release of P from 500 FTU of phytase. This hypothesis was supported by results from the second experiment, indicating that 500 FTU/kg phytase only released 0.08% STTD P. Additionally, Ca levels in the phytase containing diets used in the first experiment were not reduced, which may negatively affect phytase efficacy (Sandberg et al., 1993; Lei et al., 1994; Qian et al., 1996).

The observation that phytase supplementation improved bone ash and P on a weight basis whereas bone P on a percentage basis was not affected was in agreement with results of previous experiments (Walk et al., 2013; Santos et al., 2014; Vigors et al., 2014; Zeng et al., 2014). It is likely that bone weight is increased whereas the composition of bone ash does not change when phytase is supplemented in the diet. As a consequence, bone ash percentage is not changed by phytase supplementation, which indicates that bone ash percentage may not be as sensitive an indicator to evaluate phytase efficacy as bone ash weight.

### Effect of Phytase on Apparent Total Tract Digestibility of Energy and Nutrients

Phytate is negatively charged in aqueous solutions, especially under acidic condition, which makes the phytate molecule bind other nutrients, including starch, protein, and minerals (Noureddini and Dang, 2009). Additionally, phytate may bind to endogenous digestive enzymes, and thereby reduce their capacity to digest nutrients (Selle et al., 2006). As a result, increasing phytate levels in the diets may reduce pepsin activity in weanling pigs (Woyengo et al., 2010), and intestinal α-amylase, sucrase, and maltase activities in chickens (Liu et al., 2008). Phytase supplementation may result in a step-wise hydrolysis of the phytate molecule, thereby releasing phytate bound P and other nutrients, which may improve the digestibility of these nutrients.

Results of experiments to study phytase supplementation on ATTD of nutrients and energy have been inconsistent. Increasing the dosage of an *E. coli* phytase from 0 to 4,000 FTU/kg did not affect ATTD of dry matter and gross energy in growing pigs fed corn-soybean meal based diets (She et al., 2018), but a linear increase in the ATTD of dry matter and gross energy was observed if the dose of a different *E. coli* phytase was increased in corn-soybean meal diets fed to nursery pigs (Arredondo et al., 2019). Numerous studies with a *Buttiauxella* spp. phytase also showed conflicting results in terms of ATTD of dry matter and gross energy in nursery pigs or growing pigs, with some studies demonstrating a positive effect of phytase supplementation (Adedokun et al., 2015; Velayudhan et al., 2015; Dersjant-Li et al., 2017), whereas no effect was observed in other experiments (Liao et al., 2005a; Zeng et al., 2016; Dersjant-Li et al., 2019). Increasing dosages of *Buttiauxella* spp. phytase did not affect AID of crude fiber, neutral detergent fiber and acid detergent fiber or ATTD of crude fiber or acid detergent fiber (Zeng et al., 2016). However, a linear reduction in the ATTD of neutral detergent fiber was observed when phytase supplementation was increased from 0 to 20,000 FTU/kg in nursery pigs fed a corn–soybean meal diet. In contrast, increasing levels of *E. coli* phytase from 0 to 4,000 FTU/kg did not affect ATTD of neutral detergent fiber, but linearly increased ATTD of acid detergent fiber by nursery pigs fed corn–soybean meal diets (She et al., 2018). Results of some studies indicated that ATTD of N or crude protein was not affected by *E. coli* or *Buttiauxella* spp. phytase in nursery pigs (Zeng et al., 2014; Arredondo et al., 2019), whereas results of other experiments indicated that ATTD of N or crude protein was increased by phytase supplementation in a dose-dependent manner in weanling or growing pigs (Adedokun et al., 2015; Zeng et al., 2016; Dersjant-Li et al., 2017, 2019). To the best of our knowledge, the present results are the first to demonstrate an increase in ATTD of ether extract as phytase was included in the diets. The fact that increased ether extract digestibility did not result in improved energy digestibility may be due to the low level of ether extract in the diet. The discrepancy among different studies in terms of ATTD of energy and nutrients may be attributed to phytase source, phytase inclusion levels, diet composition, and animal age.

### Effects of Phytase on Ca and P Digestibility

Apparent or standardized total tract digestibility of Ca and P in the negative control diet was within the range of reported values in corn–soybean meal diets fed to nursery or growing pigs (Almeida et al., 2013; She et al., 2017, 2018; Arredondo et al., 2019). The increase in STTD of Ca by 500 FTU/kg phytase supplementation in the current study was 18.73 percentage units compared with the negative control diet, indicating that 500 FTU/kg phytase released 0.07 percentage units of digestible Ca. Inclusion of 500 FTU/kg of different sources of *E. coli* phytase in corn-soybean meal diets fed to nursery or growing pigs released 0.06 to 0.10 percentage units of digestible Ca (Almeida et al., 2013; She et al., 2018; Arredondo et al., 2019), although a lack of increase in Ca digestibility by microbial phytase has also been reported (She et al., 2017). The observation that STTD of Ca was maximized at 526 FTU/kg phytase supplementation is in agreement with data indicating that Ca digestibility by nursery pigs is maximized by inclusion of 574 FTU/kg to 1,041 FTU/kg (Almeida et al., 2013; Arredondo et al., 2019). These observations indicate that there may be limited Ca bound to phytate in corn and soybean meal.

The observation that 500 FTU/kg phytase released 0.08 percentage units of digestible P in the current experiment was also in agreement with data indicating that 500 FTU/kg in nursery or growing pig fed corn-soybean meal diets released from 0.05 to 0.08 percentage units of STTD P (Almeida et al., 2013; She et al., 2017, 2018; Arredondo et al., 2019). Likewise, *Buttiauxella* spp. phytase supplementation at 500 FTU/kg release 0.08% digestible P (Adedokun et al., 2015; Dersjant-Li et al., 2017). The recent meta-analysis suggests that the average release of digestible P by 500 FTU/kg phytase is 0.06 percentage units (Rosenfelder-Kuon et al., 2020). The observation that STTD of P was maximized at 834 FTU/kg phytase supplementation is also in agreement with data indicating that other sources of *E. coli* phytase maximize P digestibility at 801 FTU/kg in growing pigs (Almeida et al., 2013) and 1,107 FTU/kg in nursery pigs (Arredondo et al., 2019).

### Effect of Phytase on Ileal Amino Acid Digestibility

Commercial phytase supplementation may increase ileal AA digestibility in broilers with maximum improvement achieved at 1,000 FTU/kg (Cowieson et al., 2017a). The beneficial effect of phytase on AA digestibility improvement may be a result of reduced loss of endogenous protein in the small intestine (Woyengo and Nyachoti, 2013; Cowieson et al., 2017a). However, effects of inclusion of phytase in diets for pigs has been inconsistent (Adedokun et al., 2015; Velayudhan et al., 2015; Zeng et al., 2016; She et al., 2018) and it appears that phytase does not improve ileal AA digestibility in pigs if they are fed diets that are adequate in P (Liao et al., 2005b). Ileal AA digestibility was improved by 500 FTU/kg *A. niger* phytase only in nursery pigs fed a wheat–soybean meal-canola meal based diet, but not in pigs fed a wheat–soybean meal, a corn–soybean meal, or a barley–pea–canola meal diet (Liao et al., 2005a), which indicates that diet composition affects phytase effects on ileal AA digestibility. Overall, supplementation with up to 2,000 FTU/kg in pigs does not improve AA digestibility (Cowieson et al., 2017b), indicating that there may be species difference in terms of phytase efficacy on AA digestibility.

In conclusion, the novel *E. coli* phytase was effective in increasing growth performance, bone mineralization, and Ca and P digestibility in nursery pigs fed corn–soybean meal based diets. The maximum release of digestible Ca (0.07%) and digestible P (0.12%) were achieved at phytase inclusion levels of 526 and 834 FTU/kg, respectively. Results also indicated that this novel phytase has potential to enhance digestibility of fat and certain AA.
